# Pulmonary alveolar proteinosis: diagnostic and therapeutic challenges

**DOI:** 10.1186/2049-6958-7-4

**Published:** 2012-06-11

**Authors:** Ilaria Campo, Zamir Kadija, Francesca Mariani, Elena Paracchini, Giuseppe Rodi, Francesco Mojoli, Antonio Braschi, Maurizio Luisetti

**Affiliations:** 1 Section of Pneumology, Department of Molecular Medicine, IRCCS Policlinico San Matteo Foundation, University of Pavia, Via Taramelli 5, 27100, Pavia, Italy; 2 Section of Anesthesia and Intensive Care 1, IRCCS Policlinico San Matteo Foundation, University of Pavia, Via Taramelli 5, 27100, Pavia, Italy

**Keywords:** GM-CSF, Pulmonary alveolar proteinosis, WLL

## Abstract

Pulmonary Alveolar Proteinosis (PAP) is a rare syndrome characterized by pulmonary surfactant accumulation within the alveolar spaces. It occurs with a reported prevalence of 0.1 per 100,000 individuals and in distinct clinical forms: autoimmune (previously referred to as the idiopathic form, represents the vast majority of PAP cases, and is associated with Granulocyte-Macrophage Colony Stimulating Factor (GM-CSF) auto-antibodies; GMAbs), secondary (is a consequence of underlying disorders), congenital (caused by mutations in the genes encoding for the GM-CSF receptor), and PAP-like syndromes (disorders associated with surfactant gene mutations). The clinical course of PAP is variable, ranging from spontaneous remission to respiratory failure. Whole lung lavage (WLL) is the current standard treatment for PAP patients and although it is effective in the majority of cases, disease persistence is not an unusual outcome, even if disease is well controlled by WLL.

In this paper we review the therapeutic strategies which have been proposed for the treatment of PAP patients and the progress which has been made in the understanding of the disease pathogenesis.

## Review

Pulmonary alveolar proteinosis (PAP) is a diffuse pulmonary disease characterized by the accumulation of periodic acid-Schiff (PAS)-positive lipoproteinaceous material, primarily phospholipid surfactant and surfactant apoproteins, in the distal air spaces, which results in impaired gas transfer.

PAP is an extremely rare disorder, occurring worldwide with an estimated prevalence of 0.1 per 100,000 individuals. The clinical course of PAP is variable, ranging from spontaneous remission to respiratory failure. The onset of clinical disease is insidious, with a subacute indolent course that often delays the diagnosis by months to years. This delay is secondary to the time required for sufficient surfactant accumulation in the alveoli to impair gas exchange. In fact, decreased clearance of surfactant from the alveoli has been postulated as the basis of PAP pathogenesis.

### PAP pathogenesis

Major advancements have been achieved in the understanding of the pathogenesis in the last two decades. The first milestone was the discovery in GM-CSF knockout mice that surfactant lipids and proteins progressively accumulate in the alveolar space, as in human PAP [1,2]. These findings revealed an unexpected critical role of GM-CSF in the homeostasis of surfactant in humans. However, unbound immunoreactive GM-CSF is detectable in BAL fluid and plasma of PAP patients [3], thus researchers postulated that an inhibitory circulating factor could play a role in the pathogenesis of the disorder. With this hypothesis they laid the groundwork for the discovery of the neutralizing autoantibodies directed against GM-CSF (Abs) [4] which were proposed as the causative agent for autoimmune PAP, by impairing the function of alveolar macrophages in surfactant clearance. Later, Uchida and coworkers [5] demonstrated that in patients with PAP, the presence of GM-CSF autoantibodies was associated with impaired microbicidal activity by neutrophils, thus explaining the basis for the supposed systemic impairment of defence mechanisms in PAP. When highly purified GM-CSF autoantibodies, deriving from a patient with autoimmune PAP, were administered to healthy nonhuman primates, the pathologic manifestations of the human disorder were reproduced, thus demonstrating the causative nature of these autoantibodies [6].

### PAP classification

The vast majority of PAP (more than 90% of all reported cases) are classified as the autoimmune disease type, associated with the presence of GM-CSF autoantibodies. Less commonly is the congenital type (caused by mutations in genes coding for surfactant protein – B or –C, ABCA3, and α chain of the granulocyte-macrophage colony-stimulating factor receptor, GM-CSF-Rα) or the secondary type (forms associated with hematologic or solid malignancies, inhalation of inorganic agents, chemotherapy treatment, opportunistic infections and lysinuric protein intolerance).

When reviewing the therapeutic strategies for PAP, it is important to note that the various forms of PAP may require different treatments, even though whole lung lavage (WLL) is the current therapeutic standard.

Autoimmune PAP has been treated successfully since the early 1960s by WLL, with significant improvements in symptoms and radiographic results. In the pre-whole lung lavage era, death occurred mostly because of progressive respiratory failure and, to a lesser extent, superimposed respiratory opportunistic infections. The high frequency of respiratory infections, including those from opportunistic agents, supports the hypothesis of an impairment in lung immunity. However the presence of infections occurring also outside the lung, would suggest a systemic impairment of host defences.

Current therapy for the congenital form of the disorder depends on the patient’s age at presentation, severity of symptoms and anticipated disease course. In these cases the therapeutic approach is supportive, although successful lung transplantation has been reported.

Therapy for secondary PAP generally involves treatment of the underlying condition. For example, when the disorder is associated with a hematologic cancer, successful chemotherapy or bone marrow transplantation corrects the associated pulmonary disorder.

### WLL

The first advance in the treatment of PAP came in 1960, when Dr. José Ramirez-Rivera applied repeated “segmental flooding” as a means of physically removing the accumulated alveolar material. This “segmental flooding” provided proof that physical removal of adequate amounts of the material provided functional improvement, but as initially described, this was clearly an impractical therapy for broad application. Over the next fifty years, this procedure was sequentially refined through the routine use of general anesthesia [7,8], increased lavage volumes [9], the use of saline alone [10-12], the addition of concomitant chest percussion [13] and the successful completion of bilateral sequential WLL in the same treatment session [14]. In addition, depending on the severity of hypoxia, this procedure may be performed with the assistance of partial extracorporeal membrane oxygenation [15].

The principal criticism of this procedure is that WLL has not been standardized and no perspective clinical trials have been performed. As a result, WLL has been modified by each center [16] and a number of publications deal step by step with the technical issues [17-19]. Even if randomized clinical trials are lacking, available evidence suggests that WLL is far more effective than any other intervention.

The WLL technique at the Pavia Center is performed under general anaesthesia in an operating room or an intensive care unit. The patient is intubated with a double-lumen endotracheal tube and fibre-optic bronchoscopy is performed to confirm the appropriate tube placement. The patient is placed in the lateral *decubitus* position; the lung is lavaged in the uppermost position, while the non-lavaged lung is mechanically ventilated. 500–600 mL of warmed (37°C) saline is injected in the lung. Fluid is then collected by gravity after opening the outflow tube. Manual chest percussion may be performed to improve drainage. When the outflow, initially milky, becomes clear, chest wall percussion, which restarts and greatly enhances the removal of proteinaceous material, is added. Lavage and percussion are continued until the outflow fluid became definitively clear, which may take 3 hrs and a total of 15–20 L saline for a single lung [20].

Although there are no clearly established criteria for when to perform WLL, the recommendations indicated in available reports include: presence of persistent or progressive respiratory failure; absence of respiratory difficulty at rest, but presence of exercise desaturation (≫5% points); in selected cases, WLL may be discussed if a PAP patient, in particular a young adult, reports a significant limitation in daily or sport activities [21].

WLL is generally well tolerated. The major adverse effect of WLL is hypoxemia, especially during the emptying phase, which decreases airway pressure and increases the perfusion of the lavaged lung. Among potential complications, intraoperative resistance tends to be more common when the first lung is being lavaged. Other more common and less dangerous complications include pneumothorax, pleural effusion, and hydropneumothorax, which can be avoided by meticulous charting of the infused saline solution versus the output, and by taking care not to allow instilled fluid to exceed the fluid drained by more than a few hundred milliliters in consecutive lavages [19].

There are few contraindications, the most common is uncorrectable low oxygen saturation, convulsions and fever, which may indicate infection. Cardiopulmonary instability is a relative contraindication since WLL may lead to a fairly rapid improvement in oxygenation once the proteinaceous material is washed out of the alveoli [19]. However, over time the WLL technique has been continuously improved, thus careful assessment during the preparation and WLL, as well as the choice of the nondependent lung lavage procedure, will allow even severely impaired PAP patients to be treated successfully.

WLL improves survival, with the proportion of subjects free from recurrent PAP reaching ≫70% at 7 yrs [20]. In a long-term follow up of the Pavia series of 81 PAP patients (collected from 1989 to 2011) we found that the procedure was potentially safe and efficacious with long lasting benefits. Patients showed symptomatic, radiographic and functional improvement after whole lung lavage. Of the 47 patients lavaged, 32 underwent a single WLL, and 15 patients underwent more than one WLL. The remaining 34 PAP patients were not submitted to WLL, for different reasons (spontaneous resolution or improvement, minimal functional impairment not requiring treatment, consent refusal).

### Bronchofiberscopiclobar lavage

When WLL with generalized anesthesia may be hazardous and in paediatric patients, where the use of WLL is less well established mainly because of the technical difficulties associated with the use of large endotracheal tube, the treatment with multiple segmental or lobar lavage by fiberoptic bronchoscopy is considered safer [22-24]. The main advantage is the possibility to perform it under local anesthesia. The procedure is performed by repeated instillation of 50 mL aliquots of normal saline into a lobe until the returning fluid becomes clear. Lavage is performed in one lobe at up to six different sessions with a gap of two to three days, and the amount of lavage fluid is about 10 times less than that required for WLL.

### Exogenous granulocyte macrophage colony stimulating factor (GM-CSF) therapy

#### *Subcutaneous GM-CSF*

Although WLL is still considered the gold standard for treatment, the discovery of alveolar macrophage involvement and anti GM-CSF neutralizing antibodies led to the use of GM-CSF as a potential therapeutic approach for PAP. Since it is conceivable that the alveolar space is the site of GM-CSF signal disruption, with the impairment of surfactant catabolism in autoimmune PAP, then it is reasonable to propose that local GM-CSF supplementation would result in better treatment outcome.

Evaluation of GM-CSF augmentation as a potential therapy in autoimmune PAP was initially prompted by the demonstration of restorative activities of this cytokine on impaired surfactant metabolism and innate immunity in GM-CSF deficient mice [25]. So far, only a limited number of PAP patients have been treated with GM-CSF by subcutaneous administration. The first patient with idiopathic PAP in whom recombinant human GM-CSF subcutaneous therapy was applied, was reported in 1996 [26]. GM-CSF treatment resulted in a transient improvement in exercise capacity, and the alveolar-arterial oxygen gradient (A-aDO2).

Later, Seymour and colleagues treated 14 patients over a three month period. Patients not responding to the initial dose of 5 μg/kg/d GM-CSF underwent stepwise dose escalation until a therapeutic response (represented by improvement in oxygenation at the lung level) was obtained.

The overall response rate was 43% and lasted a median duration of 39 weeks. Exogenous GM-CSF was well tolerated, and no late side effects were reported [27].

Subsequently, in an open-label study on 25 patients, using escalating doses of GM-CSF from 5 to 18 Mg/kg/day, GM-CSF treatment was associated with amelioration of clinical and quality-of-life-related parameters only in 48%, with relapse rates of 25% among responders [28]. However this PAP series, which is the largest to date for subcutaneous therapy, considered only subjects with moderate symptomatic disease, therefore we cannot speculate on the role of this treatment in severe forms of PAP.

The results on the role of GM-CSF antibody titer as a predictor of response to GM-CSF therapy are conflicting. Two papers by Bonfield and coworkers [29,30] suggest that PAP patients with low GMAbs titer have less active disease and respond to subcutaneous GM-CSF with a further decline in autoantibodies. On the contrary, Seymour and colleagues [31] did not find correlation between serum autoantibodies and PAP severity. They suggest that delayed diagnosis, normal serum LDH levels, higher vital capacity and plasma levels of SP-B (Surfactant Protein-B) before treatment, and peak eosinophil counts following GM-CSF are markers of response to GM-CSF therapy. However, none of these are conclusive and are not accepted as prognostic variables.

### *Inhaled GM-CSF*

In a retrospective series, patients with idiopathic PAP were treated with aerosolized GM-CSF [32]. In this study, 12 PAP patients were treated with 250 mg aerosolized GM-CSF twice daily on alternate weeks for 24 weeks. All patients except one had a positive response. Two patients made a complete recovery and were disease free one and two years after discontinuing treatment. Four patients showed complete response to both the initial course or when treated again for recurrence after discontinuation of treatment. One patient required dose escalation and had a complete response. GM-CSF was well tolerated without late toxicity after a median (range) followup of 30.5 (3–68) months.

Subsequently, Tazawa et al. reported on the role of inhaled GM-CSF in 39 patients with autoimmune PAP. The study group received high-dose GM-CSF (125 μg twice daily on days 1–8 for six 2-week cycles) followed by low-dose (125 μg once daily on days 1–4 for six 2-week cycles) over a six month study period. The authors showed a response rate of 62% that was maintained in 83% patients at a one year follow up [33]. Overall these findings provide evidence that aerosolized GM-CSF is safe and effective in treating pulmonary alveolar proteinosis.

It has also been demonstrated that GM-CSF inhalation therapy decreases markers of surfactant accumulation, including total protein and SP-A (Surfactant Preotein-A) in the BAL fluid of high responders [34]. In particular, among 94 biomarkers screened, they found that the concentration of interleukin-17 and cancer antigen-125 were significantly increased after GM-CSF inhalation treatment.

Very recently, the results of a meta-analysis of observational studies [35] suggest a cumulative response rate of 59% with GM-CSF therapy in autoimmune PAP, with a trend towards better response via the inhaled route compared to the subcutaneous route. Moreover, almost 30% of the GM-CSF responders relapsed during followup; the number of relapses was less in the inhaled versus the subcutaneous group, because of the local deposition at the alveolar space which is the putative site of GM-CSF signal disruption.

The authors of this meta-analysis speculate on the theoretical possibility of inadequate deposition of GM-CSF with the inhaled route, due to the presence of surfactant in the alveolar spaces, which would not be a problem with the subcutaneous route. However, we have to keep in mind that administration of GM-CSF via the inhalation route shows the best efficacy and prevents hematopoietic toxicity, especially on the long-term basis, encountered with subcutaneous administration [36].

There is usually a lag period of 6–12 weeks in the subcutaneous treatment group and 4–12 weeks in the inhaled group prior to clinical response. This is probably due to the time required for GM-CSF to mobilize hematopoietic progenitor cells into peripheral blood and stimulate them to differentiate into functional alveolar macrophages in the lung.

The overall response rate of GM-CSF therapy is lower compared with WLL, in fact it seems only to limit disease progression. For this reason, it has been proposed as stand-alone therapy in PAP patients with less severe disease and as a supplementary therapy to WLL in patients with more advanced autoimmune PAP [36].

In this context, a decrease in GM-CSF requirements was shown by performing WLL, followed by nebulized GM-CSF [37]. It also appears that high amounts of exogenous GM-CSF can overcome the endogenous neutralizing antibodies; especially if GM-CSF is directly administered to the lung. This seems to be due to the lipoproteinaceous material acting as a barrier, which once cleared by WLL allows inhaled GM-CSF to more readily reach the alveoli.

At the Pavia center, a study protocol has been recently started with WLL followed by inhaled GM-CSF (Sargramostin) in the treatment of Autoimmune PAP (AIFA FARM7MCPK4).

### Plasmapheresis

The presence of systemic anti GM-CSF antibodies in idiopathic PAP led to the hypothesis that PAP could be an autoimmune disease and hence the rationale for plasmapheresis as a therapeutic option. A 41-year old nonsmoker woman with a 5-year history of non-resolving pulmonary infiltrates was the first case of PAP treated with plasmapheresis. She was refractory to repeated WLL sessions and subcutaneous GM-CSF. On a compassionate basis, she was treated with plasmapheresis, 1.5 plasma volume exchanges on ten separate sessions over a 2-month period. One of these episodes was complicated by gram-negative sepsis and respiratory failure. She subsequently recovered from this, and her anti–GM-CSF antibody titer was reduced, with improvement in symptoms, oxygenation, and radiographs [38]. No long-term followup data on this patient are available.

Subsequently, a patient with autoimmune PAP, presenting with persistent disease despite three WLL treatments over 10 months, was treated with plasmapheresis with ten 1.5-L plasma exchanges. Plasmapheresis lowered the serum autoantibody level, but did not improve respiratory impairment. Further WLL therapy was required, but it was transiently effective, with increased length of symptom-free periods between subsequent WLLs [39].

### Biological therapy

Reducing the autoantibody level by depleting B cells is a novel approach for PAP. Rituximab is a monoclonal antibody directed against the CD20 antigen of B-lymphocytes. It has been demonstrated to be effective in various diseases mediated by autoantibodies, such as rheumatoid arthritis, idiopathic thrombocytopaenicpurpura and systemic lupus erythematosus. Rituximab was observed to be useful in the treatment of patients with unresponsive PAP. Two single-cases have been described: Borie et al. [40] described a 41-yr-old nonsmoker who refused whole lung lavage and exhibited clinical, functional and radiographic improvement six months after rituximab therapy (1 g iv on days 1 and 15), while Amital et al. [41] described a 40-yr-old non smoker with severe dyspnoea and hypohaemia. WLL and GM-CSF initially resulted in partial remission, but a year later the patient’s condition deteriorated and only after two courses of rituximab, DL_CO_ and oxygen saturation at rest and during exercise dramatically improved.

The first prospective, open-label, proof-of-concept rituximab trial was conducted in 10 PAP patients [42]. Intervention consisted of two intravenous infusions of rituximab (1000 mg), fifteen days apart. Post-therapy followup was obtained in seven out of 10 patients. The most striking clinical finding was improvement in oxygenation. Improvements were also noted in TLC, HRCT scans and a transitional dyspnoea index. Importantly, neither the total serum anti-GM-CSF nor the serum GM-CSF neutralizing capacity were reduced following rituximab therapy. On the contrary, reduction in anti-GM-CSF levels in BAL fluid from the lung correlated with disease changes, suggesting that disease pathogenesis is related to autoantibody levels in the target organ. The data from this study also indicated that rituximab was well tolerated, with no major adverse reactions in this PAP cohort.

## Conclusions

Over the last two decades we have greatly increased our understanding of the pathogenesis of PAP. Studies in mice, in primates and lastly in humans, represent the successful pathway of translational research, by which the results obtained in animal models were quickly transferred to patients. The discovery of the critical role played by neutralizing autoantibody against GM-CSF, which causes a defect in the function of alveolar macrophages linked to the disruption of surfactant homeostasis, has led to the development of a diagnostic test with a very high sensitivity and specificity. Following this research line we will soon be able to attain a better comprehension of the biological and immunological mechanisms regulating not only PAP etiology, but also lung homeostasis.

In this context, even if a new alternative therapy to WLL has not yet emerged, modulation of the GM-CSF signaling pathway or novel biological approaches seem to be promising.

## Competing interests

The authors declare that they have no competing interests.

## Authors’ contributions

IC wrote the first draft of the manuscript; ZK, FM and EP participated in the design of the review and helped to draft the manuscript; GR, FM and AB revised the description of WLL treatment; ML coordinated the writing of the manuscript and revised substantially all the drafts. All the authors read and approved the final manuscript.
